# Evidence of the Involvement of a Plus-C Odorant-Binding Protein HparOBP14 in Host Plant Selection and Oviposition of the Scarab Beetle *Holotrichia parallela*

**DOI:** 10.3390/insects12050430

**Published:** 2021-05-10

**Authors:** Yafei Qu, Xiangyu Liu, Xu Zhao, Jianhui Qin, Yazhong Cao, Kebin Li, Jing-Jiang Zhou, Senshan Wang, Jiao Yin

**Affiliations:** 1 College of Plant Protection, Gansu Agricultural University, Lanzhou 730070, China; qyf18730281819@163.com (Y.Q.); jjzhou@gsau.edu.cn (J.-J.Z.); 2 State Key Laboratory for Biology of Plant Diseases and Insect Pests, Institute of Plant Protection, Chinese Academy of Agricultural Sciences, Beijing 100193, China; hlju_lxy@163.com (X.L.); zx18333063191@163.com (X.Z.); 17863800974@163.com (J.Q.); yzcao@ippcaas.cn (Y.C.); kbli@ippcaas.cn (K.L.); 3 State Key Laboratory of Green Pesticide and Agricultural Bioengineering, Ministry of Education, Guizhou University, Huaxi District, Guiyang 550025, China

**Keywords:** *Holotrichia parallela*, odorant-binding protein, fluorescence competitive binding, RNA interference, EAG recording, behavior response, scarab beetle

## Abstract

**Simple Summary:**

The scarab beetle *Holotrichia parallela* is a serious underground pest and causes serious damages in China to a variety of crops. To reduce the use of pesticides, insect olfactory proteins attract more and more attention in the development of pollution-free control agents in plant protection. In this study, we evaluate the molecular mechanism in the scarab beetle to detect oviposition cues. We clone a leg biased gene *HparOBP14* which encodes for an odorant-binding protein of the scarab beetle and demonstrate its involvement in binding, electrophysiological, and behavioral responses to the oviposition chemicals by the knockdown of *HparOBP14* expression using RNA interference technique. Our study provides a strong theoretical basis for the development of environmentally acceptable strategies for *H. parallela* control.

**Abstract:**

*Holotrichia parallela* is one of the agriculturally important scarab beetle pests in China. In this study, *HparOBP14* was cloned, which is the most abundantly expressed among the OBP genes in the legs of female *H. parallela* adults. Sequence comparison and phylogenetic analysis showed that HparOBP14 has a Plus-C structure motif. The expression profile analysis revealed that *HparOBP14* expression was the highest in the female antennae and then in the legs. The fluorescence competitive binding experiment of the recombinant HparOBP14 protein showed that HparOBP14 had an affinity with 6-methyl-5-heptene-2-one (plant volatile), 3-methylindole, *p*-cymene, methanol, formaldehyde, α-pinene, and geraniol (organic fertilizer volatile). Knockdown *HparOBP14* expression decreased significantly the EAG response of the injected female adults to *p*-cymene, methanol, formaldehyde, α-pinene, and geraniol. Similarly, the injected female adults were significantly less attracted to geraniol and methanol. Therefore, HparOBP14 might bind organic matter volatiles during oviposition. These results are not only helpful to analyze the olfactory recognition mechanism of female adult *H. parallela* when choosing suitable oviposition sites, but also to provide target genes for green prevention and control of *H. parallela* in the future.

## 1. Introduction

The scarab beetle *Holotrichia parallela* Motschulsk (Coleoptera: Scarabaeida e) is a seriously harmful pest in agriculture and horticulture in China. The beetles principally damage the leaves of plants such as elm, willow, and apple tree. The larvae eat the roots of peanut, sweet potato, soybean, corn, and various other vegetable crops, as well as turf and ornamental species in fields, causing more than 15% damage to crops [1,2,3,4]. As an underground pest living in soil, its concealment brings great difficulties in control. The quick and efficient measures to larval control mainly rely on the use of pesticides [4,5]. However, the frequent use and misuse of these chemicals can lead to residual toxic substances in soil, water, and food [6]. To ensure food safety, the use of pollution-free prevention methods is promoted.

One of the important pollution-free control methods is to use insect semiochemicals to trap and kill adults, then to reduce larval populations [7,8,9,10]. Insect semiochemicals can regulate behaviors of insects, such as feeding, mating, aggregation, and reproduction [11]. Among them, oviposition is the key stage that insects must go through for reproduction [12].

It has been observed that the scarab beetles prefer to lay eggs in fields where organic fertilizers are applied [13,14]. Organic fertilizers increased the larval density of the green June beetle *Cotinis nitida* in field, as a food source for larvae, by attracting female adults to lay eggs [15]. The larval number of the African black beetle *Heteronychus arator* was high in fields treated with farm manure [16]. It was found that organic fertilizers were the best attractant to the female adults of the scarab beetle *H. parallela*, and the volatiles from the organic fertilizers were able to regulate the oviposition behavior of *H. parallela* [17]. For instance, (*Z*)-3-hexen-1-ol, 3-carene, *p*-cresol, and eugenol, that was identified from organic fertilizers, were highly attractive for *H. parallela* females [17]. Besides, the volatiles from organic fertilizers such as *p*-cresol, butanoic acid, and indole was shown to play a key role in locating the oviposition site of female *H. oblita* adults [18]. It was reported that geraniol can also stimulate the oviposition of *Maruca vitrata* and increase its production of ovulation, and methanol can lure *Plutella xylostella* to oviposition [19,20].

Insects use their sensitive olfactory systems to recognize odors and to help them to survive and reproduce (e.g., host locating, choosing mates, finding oviposition sites) [21,22,23,24]. Odorant-binding proteins (OBPs) bind and dissolve odor molecules that reach the cuticle pores of insect antennae and transfer them through the sensillum to olfactory receptors (ORs) on the dendritic membrane of olfactory neurons, resulting in changing the cell membrane permeability, generating action potentials, and, finally, initiating nerve impulses that cause insects to produce corresponding behavior [25,26,27]. It has been reported in different insect species that OBPs play an important role in insect oviposition behavior. For example, CquiOBP1 of *Culex* mosquitoes participates in the recognition of oviposition pheromones (6-acetoxy-5-hexadecanolide) [28]. Silencing of the *H. oblita* OBP gene *HoblOBP24* weakened the tropism behavior of the female adults to the oviposition attractants p-cresol and indole [29].

Most research mainly focused on antennae biased OBPs, but OBPs can also be highly expressed in other tissues such as legs [30,31]. *OBP57d/e* was found to be expressed in *Drosophila sechellia* legs, and the replacement of a *OBP57d/e* region altered the oviposition behavior [32,33]. Nine *Ectropis obliqua* OBP genes that were biased to express in the female legs were thought to relate to the oviposition behavior of the females [34].

Here, we molecularly characterized an OBP gene, *HparOBP14,* which is highly expressed in female *H. parallela* legs, and investigated its binding characteristics to host plant and organic fertilizer volatiles. Finally, we studied its function using RNA interference in combination with electrophysiological and behavioral observations.

## 2. Materials and Methods

### 2.1. Insect Rearing

Adults of *H. parallela* were caught in Shijiazhuang, China, and reared in plastic boxes with soil (100 cm × 50 cm × 50 cm, each box contained 50 males and 50 females) using fresh elm leaves as food sources. The insects were maintained in the box at 18 ± 1% humidity and 25 ± 1 °C temperature.

### 2.2. Tissue-Specific RNA Extraction and cDNA Synthesis

The tissues (antennae, heads without antennae, thoraxes, abdomens, legs, and wings) of 9–11-day-old female beetles were collected separately into 1.5 mL centrifuge tubes, frozen quickly in liquid nitrogen, and then stored at −80 °C for further study. The total RNA was extracted using a total RNA extraction kit (Tianmo, Beijing, China) according to the manufacturer’s instructions. The yield and quality of RNA were determined (A260/A280 = 1.8~2.0) with NanoDrop 2000c spectrophotometer (Thermo Fisher Scientific, Waltham, MA, USA) and 1% agarose gel electrophoresis. First-strand cDNA was synthesized with FastKing gDNA Dispelling RT SuperMix (TIANGEN, Beijing, China). The reverse transcription reaction contains: 4 μL 5 × FastKing-RT SuperMix, 1 μL total RNA (100 ng/μL), 15 μL RNase-Free ddH_2_O. The condition of the reverse transcription reactions was as follows: 42 °C for 15 min (reverse transcription reaction), followed by 95 °C for 3 min (inactive enzymes). All cDNA samples were stored at −20 °C.

### 2.3. Phylogenetic Analysis and Expression Levels of OBP Genes

Twenty OBP genes were identified from results of the transcriptome of female *H. parallela* legs [35]. The gene-specific primers of *HparOBP* genes were designed by Primer Premier 5 software (PREMIER Biosoft, San Francisco, CA, USA) and are listed in Table S1. The specificity of each primer set was validated by melting curve analysis, and the efficiency was calculated by analyzing the standard curves with a fivefold cDNA dilution series. The expression level of all 20 *HparOBPs* in the legs of *H. parallela* female adults was analyzed, and the expression of *HparOBP14* in each tissue (antennae, heads without antennae, thoraxes, abdomens, legs, and wings) was analyzed using RT-qPCR with the gene-specific primers using the 7500 Real-Time PCR Detection System (ABI, Singapore). The first strand cDNAs synthesized by the reverse transcription in 2.2 described above were used as templates. The *GAPDH* (glyceraldehyde-3-phosphate dehydrogenase) was used as an internal reference gene for normalizing and calculating relative *HparOBPs* expression. According to the operation of GoTaq^®^qPCR Master Mix Kit (Promega, Wisconsin, US), the RT-qPCR reaction was prepared on ice with 2 μL of cDNA template, 10 μL of the Master Mix, 0.5 μL of each of the primer pairs (10 μmol/L), and 7 μL of ddH2O. The RT-qPCR reaction was carried out by 95 °C desaturation for 2 min, followed by 40 cycles of 95 °C for 30 s, and 60 °C for 1 min. Each reaction was performed with three biological samples as biological replicates and repeated three times as technical replicates. The data of the obtained Ct values was analyzed by using 2^−ΔΔCT^ method [36].

The phylogenetic tree of the mature OBPs was constructed by MEGA7.0 using the maximum likelihood method [37]. Bootstrap support was assessed by a bootstrap procedure based on 1000 replicates. The amino acid sequences included 13 *H. oblita* OBPs, 20 *H. parallela* OBPs, 7 *Anomala corpulenta* OBPs, 6 *Anoplophora chinensis* OBPs, 4 *Dendroctonus ponderosae* OBPs, 7 *Dastarcus helophoroides* OBPs, 7 *Colaphellus bowringi* OBPs, and 3 *Tenebrio molitor* OBPs.

### 2.4. Molecular Cloning of HparOBP14

Primer Premier 5.0 was utilized to design specific primers with Bam*HI* and Hind*III* restriction sites (Table S1). The RNA was extracted from female antennae and used to synthesize cDNA for molecular cloning. The full-length sequence of *HparOBP14* was amplified using 2 × Taq PCR Master Mix kit (TIANGEN Biotech, Beijing, China). The PCR reaction mixture contained 1 μL cDNA template, 25 μL 2 × PCR Master Mix, 1.5 μL each of forward and reverse primers (10 μmol/L), and 21 μL H2O. The PCR reaction was carried out at 95 °C for 3 min, followed by 35 cycles of 94 °C for 30 s, 60 °C for 30 s, 72 °C for 30 s, and final 72 °C extension for 10 min. The amplified products were detected by 1% agarose gel electrophoresis and purified according to the instructions of the TIANel Midi Purification Kit (TIANGEN Biotech, Beijing, China). After digestion with *Bam*HI and *Hind*III, they were ligated into pGEM-T4 Easy Vector at 4°C overnight to make pGEM-T4-OBP14 constructs. The ligation reaction mixture (5 μL) was transformed into Trans5α chemically competent cell (50 μL) and plated on agar plates (contains a final concentration of 50 μg/mL kanamycin). The positive clones (white monoclone) were randomly selected for sequencing. The sequence obtained was analyzed by BLAST search with the National Center for Biotechnology Information (NCBI) BLAST server in 10 May 2019 (http://www.ncbi.nlm.nih.gov/). The amino acid sequence was determined from the longest open reading frame found by using NCBI ORF finder in 10 May 2019 (https://www.ncbi.nlm.nih.gov/orffinder/) and compared by using DNAMAN. Signal peptide was predicted using Signalp-5.0 Server in 10 May 2019 (http://www.cbs.dtu.dk/services/SignalP/index.php).

### 2.5. Expression and Purification of Recombinant Protein

The pGEM-T4-OBP14 construct and pET-30a(+) plasmid were digested with *Bam*HI and *Hind*III at 37 °C for 4~5 h. The target fragments were gel purified and ligated with T4 DNA ligase (Takara Bio, Beijing, China) to obtain the expression construct pET30a-OBP14. The pET30a-OBP14 construct was transformed into BL21(DE3) cells (TIANGEN, Beijing, China), and the positive clone was picked and cultured in 5 mL LB medium containing kanamycin (final concentration, 50 µg/mL) overnight at 37 °C with vigorous shaking. The bacteria solution was sent out for sequencing (Shenzhen Huada Genomics Co., Ltd.) and an aliquot was also stored at −20 °C. The sequenced bacteria solution that was shown to contain the correct *HparOBP14* sequence was inoculated into 500 mL LB liquid medium (supplemented with final concentration 50 µg/mL kanamycin) at a ratio of 1:100 and cultivated at 37 °C with 220 r/min vigorous shaking until the OD_600_ value reached 0.8~1.0, then isopropyl-β-D-1-thiogalactopyranoside (IPTG) was added at a final concentration of 0.25 mmol/L. The expression of *HparOBP14* was induced for 8 h at 28 °C with 180 r/min shaking. SDS-PAGE was used to monitor the expression of the recombinant HparOBP14 protein. The protein expressed in the supernatant was purified by Ni ion affinity chromatography (ÄKTA™ Avant Cytiva, GE, USA). The eluent containing HparOBP14 protein was dialyzed overnight in a Tris-HCL buffer (50 mmol/L, pH 7.4). The His-tag of the purified recombinant protein was removed by using recombinant enterokinase (Shanghai Korain Biotech Co Ltd., Shanghai, China), then a second purification was performed with the Ni affinity chromatography. The protein concentration and purity were determined with FlexStation3 microplate reader (Molecular Devices Inc.,CA, USA), and the pure protein samples were stored at −80 °C for later use.

### 2.6. Fluorescence Displacement Binding Assay

To determine the affinity of HparOBP14 to odorant volatiles, the fluorescent displacement binding assay was carried out [36]. A total of 27 compounds was selected, including 7 compounds from host plant (elm) volatiles [38,39], 11 compounds from organic fertilizer volatiles [17], and 9 compounds common in both of them. The odorant compounds (purity ≥95%) were purchased from Sigma-Aldrich. The fluorescent probe N-phenyl-1-naphthylamine (1-NPN) was dissolved in methanol (chromatographic grade) to a final concentration of 1 mM and used as the stock solution. The stock solution of the purified protein was diluted with Tris-HCl (50 mmol/L, pH 7.4) to a final concentration of 2 μM and added into a colorimetric plate cuvette. The 1-NPN stock solution was then successively added to the protein solution to the final concentrations ranging from 2 μM to 20 μM. The fluorescence changes were recorded to get K_1-NPN_, the dissociation constant of the complex HparOBP14/1-NPN.

The binding ability of HparOBP14 to the odorant compounds was determined by titrating the mixture of the protein and the 1-NPN (final concentration 2 μM of both) with each odorant compound at final concentrations ranging from 2 μM to 40 μM. The competitive dissociation constant was calculated from the corresponding IC_50_ values using the following equation: Ki = [IC_50_]/(1+ [1-NPN]/K_1-NPN_), where [1-NPN] is the free concentration of 1-NPN; K_1-NPN_ is the dissociation constant of the complex HparOBP14/1-NPN.

The fluorescence changes during the binding of either 1-NPN or odorant compounds to the HparOBP14 protein were measured and recorded with the excitation wavelength of 337 nm and within a wavelength range of 350~550 nm by a 970CRT fluorescence spectrophotometer (Yidian Analytical Instrument Company, Shanghai, China).

### 2.7. RNAi-Mediated Gene Silencing

The dsRNAs of *HparOBP14* and *GFP* (GenBank number: ACY56286) (for primers, see Table S1) were synthesized using a T7 Ribo MAX™ Express RNAi System (Promega, Madison, WI, USA) according to the manufacturer’s instructions. The GFP-dsRNA was used as the negative control. Nine-to-eleven-day-old female adult beetles were injected with 2 μg dsRNA into the adult head with a 5 μL injection needle (7000 series syringes, Hamilton, USA), then kept under the same condition as 2.1 described above. The antennae were dissected at 24 h, 48 h, 72 h, and 96 h after injection, and the samples were stored at −80 °C for further RT-qPCR analyses.

### 2.8. Electroantennogram (EAG) Recording

The EAG recording was conducted on the adult antennae with the EAG micromanipulator MP-15 and analyzed with EAG-Pro (Syntech, Kirchzarten, Germany). Briefly, two ends of the antennae were set on electrode holders with a conductive adhesive (Spectra 360 electrode gel). An ashless filter paper (Hangzhou Special Paper Co., Ltd., Hangzhou, China) was cut into strips (3 cm × 0.5 cm). A 20 μL compound solution (concentration 1 μg/μL in paraffin oil) was applied onto the paper strip which was then placed in a Pasteur tube (17 cm × 12 cm) and applied to the antennae by purified air at a flow rate of 1 L/min. The paraffin oil (20 μL) was used as a control. The stimulus recording time was 0.5 s and the interval between two stimulations was more than 1 min.

### 2.9. Y-Tube Olfactometer Behavioral Bioassays

The Y-tube olfactometer mainly consisted of a Y-shaped glass tube (main arm, 30 cm; sidearm, 20 cm; inner diameter, 2.5 cm; side arm angle, 60 degrees), an air sampler, a drying tower, a gas scrubber, and a flow meter. Two gas-washing cylinders were successively connected with the flow meter, drying tower, air pump, and Y-shaped glass tube through rubber pipes. The testing compound (concentration 1 μg/μL in paraffin oil) and paraffin oil (as control) were taken and dropped respectively into a rubber septum (Pherobio Technology Co., Ltd., Beijing, China) and placed respectively into the side arms of the Y-tube. The flow rate of the atmospheric air sampler was adjusted to 500 mL/min.

A female adult beetle was introduced into the main arm at the downwind end of the Y-olfactometer. Each beetle was given 5 min to respond to the treatment, and the first choice that the beetle made for each of the lateral arms was recorded. The response was regarded as valid only if the beetle crawled more than 3 cm into a lateral arm and stayed for more than 1 min. The following measurements were recorded for all individual beetles: the number of beetles that crawled into a lateral arm of the Y-tube (responsive beetles), and the number of beetles that did not make any choice and stayed in the main arm for 5 min (non-responsive beetles).

For each compound, more than 90 beetles were tested individually until 90 responsive beetles were obtained. Each individual beetle was tested only once, and the Y-olfactometer was replaced after every 10 beetles were tested to eliminate the odor effect. The number of individual beetles that selected the compound-treated arm of the Y-olfactometer was statistically compared with the number of beetles that selected the solvent-treated arm using a Chi-square goodness of fit test.

### 2.10. Statistical Analysis

The data of *HparOBP14* expression in different tissues was analyzed by one-way analysis of variance (ANOVA) followed by Tukey’s multiple comparison test for the comparison of the means with the SPSS Statistics version 23 (IBM Corp., Armonk, NY, USA). The data of behavioral, electrophysiological experiments, and RNAi treatment were analyzed by independent-sample t-test. All figures were generated with Prism 8 (GraphPad, La Jolla, CA, USA).

## 3. Results

### 3.1. Phylogenetic and Expression Level Analysis of 20 HparOBP Genes

**Figure 1:**
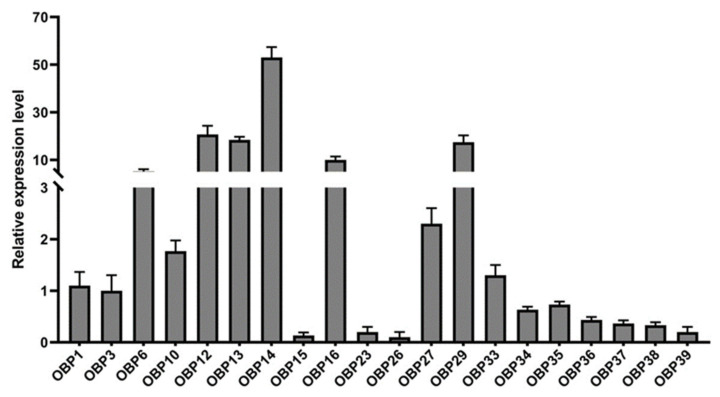
The expression patterns of different *HparOBP* genes in female legs. The fold changes are relative to the transcript levels in the *OBP1*. The *GADPH* gene was used as reference to normalize the expression of each tested gene. The data are presented as mean ± SEM and the error bars represent the standard error (n = 3). The Y-axis is broken to better indicate the low-level expression of some genes.

RT-qPCR method was used to verify the differential expression of 20 *HparOBP* genes in the legs of female *H. parallela* adults. Relative to the expression of *HparOBP1*, *HparOBP14* was the most highly expressed OBP gene in the legs, and the expression of six other genes (*HparOBP6*, *HparOBP12*, *HparOBP13*, *HparOBP16,* and *HparOBP29*) was also relatively high (Figure 1). Therefore, *HparOBP14*, the most highly expressed OBP gene in the legs, was selected for subsequent studies.

**Figure 2:**
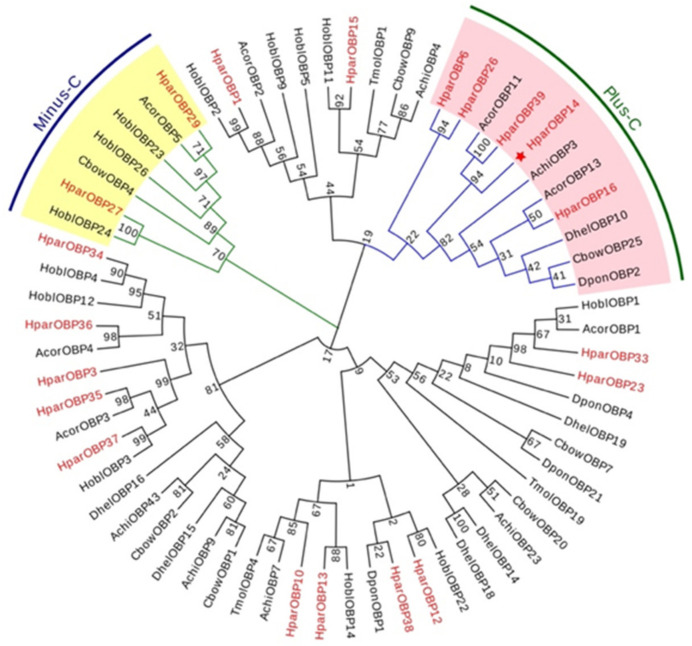
Phylogenetic tree of HparOBP14 and OBPs from other insect species. The OBPs of *Anomala corpulenta* (Acor), *Holotrichia oblita* (Hobl), *Anoplophora chinensis* (Achi), *Dendroctonus ponderosae* (Dpon), *Dastarcus helophoroides* (Dhel), *Colaphellus bowringi* (Cbow), and *Tenebrio molitor* (Tmol) were used to construct the phylogenetic tree with MEGA7.0 using the maximum likelihood method and 1000 bootstrapping. The numbers are the bootstrapping values for the branch.

The mature protein sequences of 20 *HparOBP* genes were compared with 46 OBPs of Coleoptera in the phylogenetic tree constructed using MEGA 10.0 software by maximum likelihood (ML) method with 1000 bootstrap replicates (Figure 2). In the phylogenetic analysis, HparOBP14 with HparOBP6, HparOBP16, HparOBP26, HparOBP39, AcorOBP11, AcorOBP13, DponOBP2, DhelOBP10, AchiOBP3, and CbowOBP25 all belong to Plus-C OBPs (Figure 2, pink area). HparOBP14 is clustered with AcorOBP11and HparOBP39 in the same branch, indicating that they are closest to each other. HparOBP27, HparOBP29, HoblOBP23, HoblOBP24, HoblOBP26, AcorOBP5, and CbowOBP4 all belong to Minus-C OBPs (Figure 2, yellow area). The analysis shows that 66 OBPs have high diversity, and there are only a few obvious homologous groups in the same species.

### 3.2. Molecular Cloning HparOBP14

The full-length cDNA encoding *HparOBP14* (GenBank: KR733560.1) [40] was cloned and verified by sequencing. The *HparOBP14* sequence contains a single open reading frame (ORF) of 627 nucleotides and encodes a protein of 208 amino acids with a signal peptide of 17 amino acids at the N-terminal (Figure S1). The predicted molecular weight is 23.8 kDa. HparOBP14 and *Anomala corpulenta* OBP11 (GenBank: AKC58532.1) has the highest similarity of 49.06%, while it has a high similarity with *H. parallela* OBP16 (GenBank: AKI84374.1) and *Anoplophora chinensis* OBP3 (GenBank: AUF7295 0.1), with a similarity of 32.29% and 29.44%, respectively (Figure S2). HparOBP14 has the characteristics of the Plus-C OBP subgroup of the insect OBP family. In addition to the six highly conserved cysteine residues in typical Classical OBPs, HparOBP14 has two conserved cysteine residues in the region before C1. Between C4 and C5, and after C6, there are two additional conserved cysteine, C4a and C6a. A conserved proline is located downstream of C6 (Figure S2). The conserved motif was of HparOBP14 was C1-X24-C2-X3-C3-X43-C4-X12-C4a-X9- C5-X8-C6-P-X9-C6a-X21 (X is any amino acid followed by the numbers of amino acids) [41].

### 3.3. Tissue Expression Profile Analysis of HparOBP14

**Figure 3:**
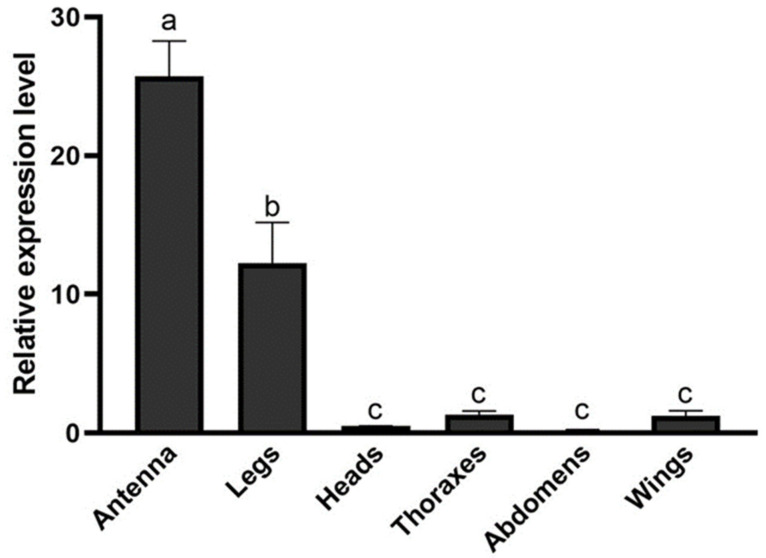
Relative expression of *HparOBP14* gene in the tissues of female *H. parallela*. Relative fold changes were normalized to the transcript levels in female abdomen. The GADPH gene was used as reference to normalize the expression of each tested gene. Error bars represent the standard error (n = 3). The different letters (a,b,c) indicate significantly difference (ANOVA followed by Turkey’s HSD multiple comparison test, *p* < 0.05).

The expression level of *HparOBP14* in different tissues of *H. parallela* was analyzed by RT-qPCR. *HparOBP14* had the highest expression in the antennae, followed by an intermediate expression in the legs and little expression in other tissues (Figure 3). The specific expression of *HparOBP14* in the antennae and legs of female adult suggests that it may play a role in some physiological functions involving antennae and legs of *H. parallela*.

### 3.4. Purification of HparOBP14 Protein

**Figure 4:**
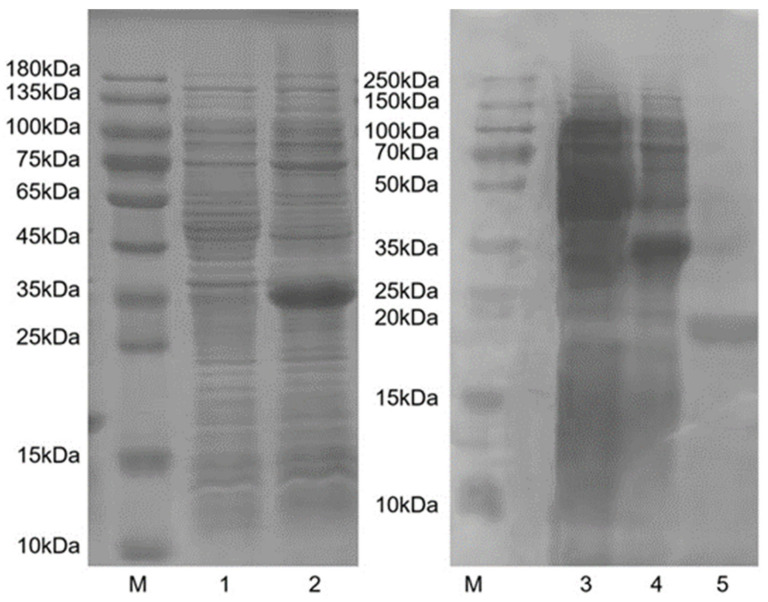
Analysis of the expression of HparOBP14 fusion protein by SDS-PAGE. M: protein molecular maker; lanes 1 and 3: bacteria samples without IPTG induction; lanes 2 and 4: bacteria samples with IPTG induction; lane 5: purified HparOBP14 protein after the removal of His-tag.

The recombinant HparOBP14 protein was expressed in the bacterial competent cell BL21 (DE3) for recombinant protein purification. Compared with the uninduced control, overexpressed protein bands were observed around 35 kDa in the bacterial solution induced by IPTG (Figure 4: lane 2 and 4). To avoid the interference on ligand binding, the His-tag was cut off with a recombinant enterokinase, and the expected single protein band around 20–25 kDa was produced (Figure 4: lane 5), which is consistent with the predicted theoretical molecular weight, 23.8 kDa, of HparOBP14 protein.

### 3.5. Fluorescence Competitive Binding Assays

**Figure 5:**
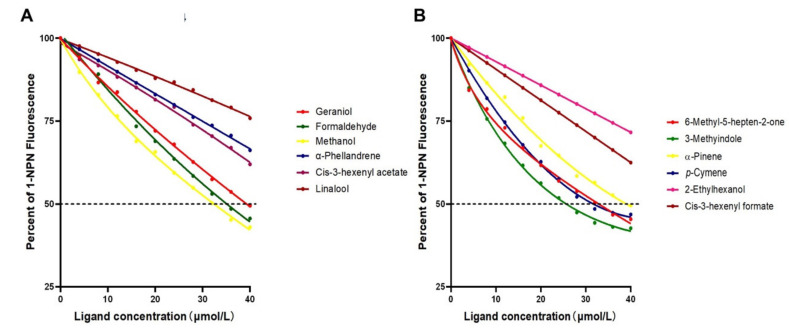
Competitive binding curves of HparOBP14 to various odorant compounds. The protein (2μM) and the Figure 1. NPN (2μM) mixture was titrated with volatiles compounds. Binding ability of protein HprOBP14 to 12 ligands (geraniol, formaldehyde, methanol, α-phellandrene, *cis*-3-hexenyl-acetate, linalool, 6-methyl-5-hepten-2-one, 3-methyindole, α-pinene, *p*-cymene, 2-ethylhexanol, and *cis*-3-hexenyl-formate) was calculated by the percentage decrease of 1-NPN fluorescence value.

The binding affinity of HparOBP14 protein to 28 different volatile compounds was determined. They include organic volatiles: 3-methylindole, *p*-cymene, α-pinene, geraniol, methanol, formaldehyde, and host plant volatiles: 6-methyl-5-heptene-2-one. Based on previous studies, N-phenyl-1-naphthylamine (1-NPN) was selected as the fluorescence probe for the binding analysis. The dissociation constant of HparOBP14/1-NPN complex is 5.8 µmol/L (Figure S3). Seven tested compounds could be bound to HparOBP14, and when the final concentration of the compound was 40 µmol/L, the fluorescence intensity of the HparOBP14/1-NPN complex decreased to less than 50% (Figure 5), suggesting a displacement of 1-NPN from HparOBP14 binding site by the compounds. The values of Ki were calculated as 20.99 µmol/L, 25.50 µmol/L, 32.83 µmol/L, 34.10 µmol/L, 27.37 µmol/L, 29.49 µmol/L, and 28.21 µmol/L, respectively, for the organic volatiles 3-methylindole, *p*-cymene, α-pinene, geraniol, methanol, formaldehyde, and for the host plant volatiles, 6-methyl-5-heptene-2-one (Table 1). The HparOBP14 had weaker, or no, binding ability with other odor compounds such as α-phellandrene, *cis*-3-hexenyl formate, *cis*-3-hexenyl acetate, linalool, and 2-ethylhexanol (Figure 5).

### 3.6. Transcription Level of HparOBP14 after dsRNA Injection

**Figure 6:**
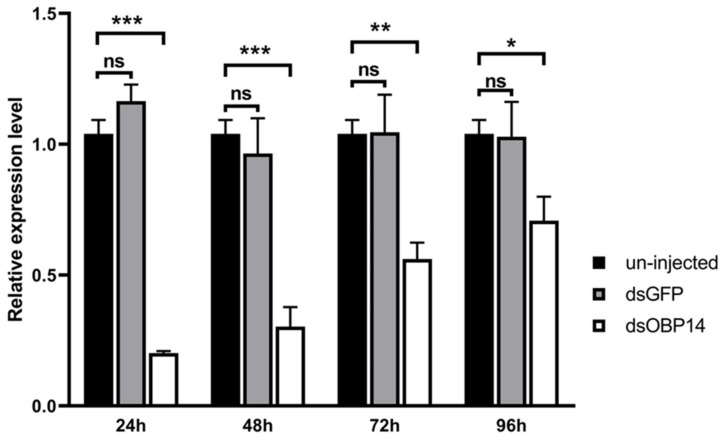
The mRNA levels of *HparOBP14* at 24 h, 48 h, 72 h, and 96 h after the RNAi treatment. RNA from antennae was used for the analysis. The GADPH gene was used as reference to normalize the expression of *HparOBP14*. Error bars represent the standard error (n = 3). The results (mean ± SEM) were evaluated using a 2^−ΔΔCT^ method, and relative to the expression level of *HparOBP14* in the female abdomen. The comparison was analyzed by independent-sample t-test (n = 3). The significant difference was presented by the stars on the top of the error bars (* for *p* < 0.05, ** for *p* < 0.01, *** for *p* < 0.001 and ns for no significant difference).

RNAi by *dsRNA* injection is an important means to examine gene functions. In this study, the expression of *HparOBP14* mRNA was detected at 24 h, 48 h, 72 h, and 96 h after *dsHparOBP14* injection. The relative expressions of *HparOBP14* in the female antennae were decreased to 20% (24 h), 30% (48 h), 56% (72 h), and 70% (96 h) by the *dsHparOBP14* injection, which are significantly different from the *GFP* gene expressions by the dsGFP injection (*p* < 0.05) (Figure 6). There was no significant change in the gene expression levels of *HparOBP14* in the antennae between uninjected and dsGFP-injected insects (*p* > 0.05) (Figure 6).

### 3.7. Electroantennogram Responses of Female H. parallela after dsRNA Injection

**Figure 7:**
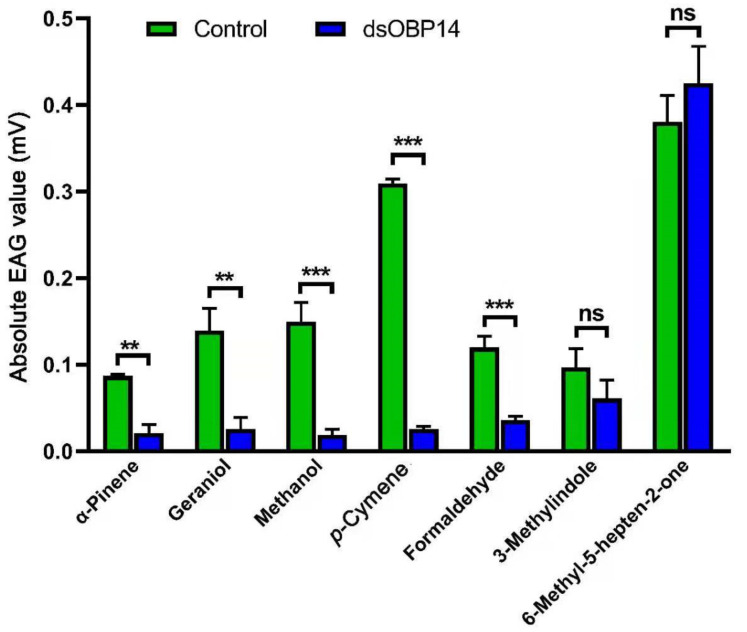
EAG response of *H. parallela* female adults. The female beetles were tested 48 h after the RNAi treatment. Error bars represent the standard error (n = 3). The data was analyzed by independent-sample t-test (n = 3). The comparison between uninjected (control) or *dsHparOBP14*-injected insects was analyzed by independent-sample t-test (n = 3). The significant difference was presented by the stars on the top of the error bars (** for *p* < 0.01, *** for *p* < 0.001 and ns for no significant difference).

The female *H. parallela* adults injected with *dsHparOBP14* for 48 h were used to measure their responses to a total of seven compounds (3-methylindole, *p*-cymene, α-pinene, geraniol, methanol, formaldehyde, and 6-methyl-5-heptene-2-one) in electrophysiological studies (Figure 7). The EAG responsive amplitudes to *p*-cymene, α-pinene, geraniol, methanol, and formaldehyde decreased significantly in the *dsHparOBP14*-injected insects (*p* < 0.05) compared to those of uninjected control insects. There are clear responses to 3-methylindole and 6-methyl-5-heptene-2-one, but their amplitudes do not change significantly after dsRNA injection (*p* > 0.05) (Figure 7).

### 3.8. Effect of dsRNA Injection on the Behavior of H. parallela

**Figure 8:**
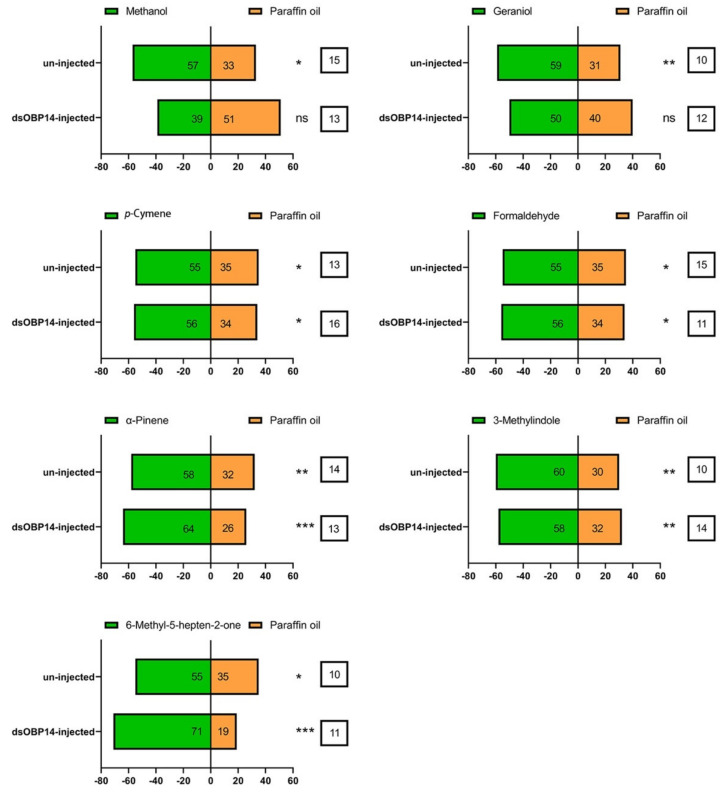
Behavioral response of female *Holotrichia parallela* adults to seven compounds. Seven compounds are α-pinene, geraniol, methanol, *p*-cymene, formaldehyde, 3-methylindole, and 6-methyl-5-heptene-2-one. The numbers in the green bars indicate the number of females that made the choice to the Y-tube olfactometer arm treated with odor compounds. The numbers in the orange bars indicate the number of females that made the choice to the Y-tube olfactometer arm treated with the solvent paraffin oil (control). The numbers in the white bars indicate the number of females that did not make any choice and stayed in the main arm for 5 min. The significant difference in the numbers between two lateral arms was indicated by * (*p* < 0.05), ** (*p* < 0.01), *** (*p* < 0.001), and analyzed with a Chi-square goodness of fit test.

The role of *HparOBP14* in *H. parallela* female adults was further verified through the Y-tube behavior experiment (Figure 8). The uninjected female beetles were attracted to the methanol, *p*-cymene, formaldehyde, 6-methyl-5-heptene-2-one (*p* < 0.05), α-pinene, geraniol, and 3-methylindole (*p* < 0.01). The attraction of the beetles to geraniol and methanol was markedly reduced by the injection of *dsHparOBP14*. The response of *dsHparOBP14*-injected female adults to α-pinene and 6-methyl-5-heptene-2-one was significantly increased (*p* < 0.001), but the attraction to *p*-cymene, 3-methylindole, and formaldehyde had no obvious change (Figure 8).

## 4. Discussion

The choice of the host plant by an insect female is a complex sequence that ends in oviposition, and it is a multi-modal behavior that involves, among other things, volatile and non-volatile chemical stimuli. The binding of these odor molecules to OBPs is an important step in the process of insect recognition of host plants and oviposition sites [37,42,43].

In this study, *HparOBP14* was found highly expressed in the legs of *H. parallela* female adults among all 20 OBP genes. *HparOBP14* is also highly expressed in the antennae of *H. parallela*. The two organs are well-known to be involved in different stages of reproductive behavior: antennae, main organs housing olfactory receptors for olfactory recognition of volatile plant compounds (VPCs), the legs carrying contact chemoreceptors allowing the recognition of non-volatile molecules once the female landed on the plant [44]. Our results show very clearly that HparOBP14 is involved in the responses of *H. parallela* female adults to the volatile compounds of the host plant or from the environment. The binding experiments indicate a low specificity of HparOBP14, the chemical structures of tested compounds being quite different. The olfactometer choice tests reveal a positive choice for the compounds tested upon female beetles. The EAG response of individual *H. parallela* female antennae to *p*-cymene, α-pinene, geraniol, methanol, and formaldehyde, but not to 6-methyl-5-hepten-2-one, was significantly reduced after HparOBP14 expression was knocked down. These results constitute an argument in favor of HparOBP14′s role in the recognition of the host plant volatiles.

It was reported that *AlinOBP11* is the most abundant gene in the forelegs of *Adelphocoris lineolatus* adults, which bound to non-volatile plant secondary compounds and had an important function in taste [45,46]. However, in our EAG and Y-olfactometer experiments, the stimulus molecules were delivered to the insects by an air flow. Thus, these experiments can only assess the involvement of HparOBP14 in the perception of volatile molecules from host plants and oviposition sites. The data do not explain HparOBP14’s function in the detection of non-volatile molecules by the legs.

Nevertheless, insect oviposition is mediated by attractive chemical cues emitted from oviposition sites in the environment [47]. Previous studies showed that the OBPs that are highly expressed in the legs of female insects play a role in the perception of plant volatiles, a behavioral step leading to oviposition [48]. *OBP30* expressed in the legs of female *Chilo suppressalis* was speculated to be associated with oviposition-related compounds [49]. *OBP57d* and *OBP57e* expressed in *Drosophila* legs could recognize the oviposition-related volatile octanoic acid [50]. Research on the oviposition preferences found that the female beetles (*Cyclocephala borealis*, *C. lurida*, *C. pasadenae*, *C. hirta,* and *C. parallela*) prefer to lay eggs in soil that is rich in organic matters [51]. In this study, the leg-biased HparOBP14 had a relatively high affinity to methanol, geraniol, 3-methylindole, *p*-cymene, α-pinene, and formaldehyde, which are chemical cues for locating oviposition sites by *H. parallela* females [17]. Furthermore, knockdown of *HparOBP14* expression significantly affected the behavior of *H. parallela* to decomposed organic fertilizer volatiles, in agreement with previous studies on the oviposition preferences of beetles [17,18,48]. The attractive response of *H. parallela* female adults was significantly decreased to methanol and geraniol by the *HparOBP14* interference. Methanol and geraniol are volatile organic compounds emitted during the decomposition of plant litter and are also the potential volatiles to attract female *H. parallela* during oviposition [17,52]. Methanol is attractive not only to *H. parallela*, but also to the root-eating beetle *Rhizophagus ferrugineus* and *H. oblita* [18,53]. Geraniol, as a compound in bait, is attractive to both sexes of the Japanese beetle *Popillia japonica* [54,55] and to the leaf beetle *Monolepta hieroglyphica* as key plant volatiles [56,57]. These results support that HparOBP14 may play a role in oviposition behaviors of *H. parallela* female adults.

It is noted that there are differences between the results of the EAG experiments and those of the behavioral experiments. This may be due to different doses used in these two experiments. It is also noted that the in vitro binding results (Table 1) are different from those of the EAG and behavioral experiments. This could be due to the limitation of the fluoresce competitive binding assay used, namely 1-NPN displacement. It is not certain whether 1-NPN could bind only into the HparOBP14 binding pocket. The compounds tested could only displace some unspecific 1-NPN binding as the affinities obtained in this study are not as good as those (<10 µM) in other studies. Furthermore, the recombinant HparOBP14 also binds the host plant volatile 6-methyl-5-heptene-2-one, and the knockdown of *HparOBP14* expression increased the attraction of the beetles to it but did not affect the EAG response of the female adults to this volatile compound. These responses could be mediated by several olfactory proteins; some of them could play a more important role than HparOBP14 in the response to 6-methyl-5-hepten-2-one. Therefore, HparOBP14 may be essential for female *H. parallela* to detect methanol and geraniol and may play a role in the process of finding suitable oviposition sites, as well as may participate in the process of identifying host plants. We cannot attribute a specific function (olfaction or taste) to HparOBP14 from the current study, but provide some evidence for the possible involvement of HparOBP14 in the perception of plant volatiles and oviposition.

## 5. Conclusions

In this study, we preliminarily identified the function of *HparOBP14* which is highly expressed in the legs of female *H. parallela*. Based on the transcriptome data, we cloned the *HparOBP14* gene and further described the tissue-specific expression and the binding spectrum as well as the changes of the electrophysiological and behavioral responses after the knockdown of *HparOBP14* expression. These results provide the evidence that HparOBP14 may be involved in the oviposition behavior of *H. parallela* female adults, and also provide a theoretical basis for the research and development of environmentally friendly pest control strategy, which may ultimately help to reduce the reproduction of *H. parallela*.

## Figures and Tables

**Table 1:** Binding of different compounds to recombinant OBP14 of *H. parallela*.

HparOBP14			
Compound	IC_50_ (μmol/L)	Int (%)	K_i_ (μmol/L)
6-methyl-5-hepten-2-one *	32.04	61.66	28.21
octanal *	-	-	-
dodecyl aldehyde *	-	92.42	-
3-hexanol *	-	84.27	-
cis-3-hexenyl formate *	-	81.25	-
cis-3-hexenyl acetate *	-	81.43	-
2-ethylhexanol *	-	85.80	-
decanal ^&^	-	77.88	-
linalool ^&^	-	89.20	-
β-caryophyllen ^&^	-	-	-
(Z)-3-hexen-1-ol ^&^	-	96.53	-
1-nonanal ^&^	-	69.22	-
α-pinene ^&^	38.50	67.49	32.83
3-carene ^&^	-	68.52	-
camphene ^&^	-	83.88	-
α-phellandrene ^&^	-	83.00	-
1-hexanol ^§^	-	72.19	-
1-heptanol ^§^	-	83.41	-
eugenol ^§^	-	86.03	-
p-cresol ^§^	-	91.23	-
indole ^§^	-	82.23	-
3-methylindole ^§^	24.61	56.22	20.99
butyric acid ^§^	-	99.07	-
geraniol ^§^	39.98	72.07	34.10
p-cymene ^§^	29.90	62.70	25.50
formaldehyde ^§^	34.58	68.91	29.49
methanol ^§^	32.10	65.66	27.37

Note: The mixtures of HparOBP14 protein and 1-NPN (2 µM) were titrated with different compounds with concentrations of 2–40 μM. Int (%): relative fluorescence intensity of 1-NPN/HparOBP14 complex after the addition of testing compound; -: could not be calculated (no binding); *: volatiles of host plants; &: volatiles from both host plants and rotten organic matters; §: volatiles from rotten organic matters.

## Data Availability

The authors confirm that the data supporting the findings of this study are available within the article and its Supplementary Materials.

## Supplementary Materials

The following are available online at https://www.mdpi.com/article/10.3390/insects12050430/s1, Table S1. Primer pairs used in this study. Figure S1. Nucleotide sequence and deduced amino acid sequence of *HparOBP14*. Figure S2. Alignment of the deduced amino acid sequence of HparOBP14 with homologous proteins from other OBPs. Figure S3. Binding curve and Scatchard plots of HparOBP14 protein with the fluorescent probe 1-NPN.
