# Supplementary material for: Evidence of the Involvement of a Plus-C Odorant-Binding Protein HparOBP14 in Host Plant Selection and Oviposition of the Scarab Beetle Holotrichia parallela
Source: Insects. 2021 May 10;12(5):430. doi: 10.3390/insects12050430 (PMC8151400; doi:10.3390/insects12050430)
Supplement: Supplementary file 1 [file insects-12-00430-s001.zip › insects-1197264-supplementary.pdf]

Table S1. Primer pairs used for the manuscript

Name	Primer sequences
HparOBP1 (RT-qPCR)	Forward: 5’-TGTGTGGTAGCTTGCGTAAA-3’<br>Reverse: 5’-TGTTACATTCGTCGCTATCG-3’
HparOBP3 (RT-qPCR)	Forward: 5’-TGCATAAGGGAATCTGGAAT-3’<br>Reverse: 5’-AGAGTCAGCGCACCCCTCCA-3’
HparOBP6 (RT-qPCR)	Forward: 5’-AATGCGTACTGGTGTTGGAA-3’<br>Reverse: 5’-CTCGCAACTATCATCATCAC-3’
HparOBP10 (RT-qPCR)	Forward: 5’-TCATAGATAAGGCCGACGCA-3’<br>Reverse: 5’-TTGCACAGATCGAGAATCGC-3’
HparOBP12 (RT-qPCR)	Forward: 5’-TTCATGCCTCTTCAAAAAAT-3’<br>Reverse: 5’-TATGGCTGGGCGTTTTAGAT-3’
HparOBP13 (RT-qPCR)	Forward: 5’-GATTACGGCGTATTTGCG-3’<br>Reverse: 5’-TCGTAAGCTGTTTCACATGA-3’
HparOBP14 (RT-qPCR)	Forward: 5’-TCCAGGAGCTGTTGCTATAATG-3’<br>Reverse: 5’-TTAATGGTCCTCTGATTTGTGAA-3’
HparOBP15(RT-qPCR)	Forward: 5’-GGGAAAGGTAGGTGCGTC-3’<br>Reverse: 5’-CCTCCAAGGTCTTCAAAGTG-3’
HparOBP16 (RT-qPCR)	Forward: 5’-TATGCGTTGCAGAATGTATG-3’<br>Reverse: 5’-CGCTTCTTTCGCTTCTGCTA-3’
HparOBP23 (RT-qPCR)	Forward: 5’-ACAGTGAAGGATCAAAAGGG-3’<br>Reverse: 5’-TGGATTGGCACCAGGTATTA-3’
HparOBP26 (RT-qPCR)	Forward: 5’-CCGATGAGTGTTTGCAAGAG-3’<br>Reverse: 5’-CAAAGTTTACAGAAACGCCA-3’
HparOBP27 (RT-qPCR)	Forward: 5’-GTTCCTCGTTTGCGTCTACA-3’<br>Reverse: 5’-GTCACCAAATACGCGTTGTC-3’
HparOBP29 (RT-qPCR)	Forward: 5’-ACTGTCTCAAATCCAGCGAA-3’<br>Reverse: 5’-CGAAGTCGTAAAAGTCCAAG-3’
HparOBP33 (RT-qPCR)	Forward: 5’-TGGAAGAATTAGCCAAACAA-3’<br>Reverse: 5’-TGGTTCTGCTTTCGCTTTAT-3’
HparOBP34 (RT-qPCR)	Forward: 5’-GCCATGTTGACTACGATTCA-3’<br>Reverse: 5’-AAACATTTGCAAATTTCATG-3’
HparOBP35 (RT-qPCR)	Forward: 5’-TTGTCACTGCGCTTTGGGTA-3’<br>Reverse: 5’-GTCAACCCTTCTCCCGCATT-3’
HparOBP36 (RT-qPCR)	Forward: 5’-CTTGCAAAACAAAAGGGAAA-3’<br>Reverse: 5’-AGGCGGTAAAATAGCGGA-3’
HparOBP37 (RT-qPCR)	Forward: 5’-TGCCTGGCGACTATGAAACT-3’<br>Reverse: 5’-ACGCACTTGAAGAAAGCATA-3’
HparOBP38 (RT-qPCR)	Forward: 5’-TTGCTCGAGGATGAAAGTGA-3’<br>Reverse: 5’-CAAGCACTTATCCTCGTTCT-3’
HparOBP39 (RT-qPCR)	Forward: 5’-AGTCCGTTGCCTGGCTGG-3’<br>Reverse: 5’-CGGGAATTGATTATGCGTTG-3’
GADPH	Forward: 5'-AATACCTTTTAGTGGTCCTTCCG-3'<br>Reverse: 5'-TGCATGCTATCACAGCTACGC-3'

HparOBP14 (cloning)	Forward: 5’-CGGGATCCTATG ATTTTGAAGATCACGCAT-3’<br>Reverse: 5’-CCCAAGCTTCA CCACCTATACGTTGTTAG-3’
dsOBP14	Forward: 5’-*GGATCCTAATACGACTCACTA TAGG*ATTTTGAAGATCACGCATATAAT-3’<br>Reverse: 5’-GGATCCTAATACGACTCACTA TAGGGACATTCCAGTTGTATTTCTTTC-3’
dsGFP	Forward: 5’-*GGATCCTAATACGACTCACT ATAGG*CTACCAGCCAAATCTAAACG-3’<br>Reverse: 5’-GGATCCTAATACGACTCAC TATAGGGCTAACCGAGAAAAGATGAC-3’

*Bam*HI and *Hin*dIII restriction sites marked with double underline. *GGATCCTAATACGACTCACTATAGG* is the enhancer and T7 promoter sequence, which is required for dsRNA synthesis.

Supplementary Figures

```
1   ATGAAGTTCGTTATTGTTCTATTATTATCTGCCCTTGCATTTATCAATGCTTATGATTTT
1    M  K  F  V  I  V  L  L  L  S  A  L  A  F  I  N  A  Y  D  F
61  GAAGATCACGCATATAATGAACTATTAGTTGAACAATATTCTCTCGCCCATCCAAGACTA
21   E  D  H  A  Y  N  E  L  L  V  E  Q  Y  S  L  A  H  P  R  L
121 CGAAGAGCCGCAGAAAATAACTGCATAGATTCCAGGAGCTGTTGCTATAATGATGCCGTA
41   R  R  A  A  E  N  N  C  I  D  S  R  S  C  C  Y  N  D  A  V
181 CCACAATATCACGCTAATGAAACAGAAGAGTGCTCGAAAGAACTAGGTTTCGATCGTTCA
61   P  Q  Y  H  A  N  E  T  E  E  C  S  K  E  L  G  F  D  R  S
241 CAAATCAGAGGACCATTAACTGATGCACAAATAAATCAAATTAAATGTCTTGCTGAATGT
81   Q  I  R  G  P  L  T  D  A  Q  I  N  Q  I  K  C  L  A  E  C
301 ATTTCCAAGAAGAAGGGACATTTCGACGCCGATGGTAACCTATTGAAAGATGAACTGTTG
101  I  S  K  K  K  G  H  F  D  A  D  G  N  L  L  K  D  E  L  L
361 AAGGATATCAGGAAACCCTTAGAACAGGTAGCGTGGTTGAAGCCAAAAGTTGAGGATATA
121  K  D  I  R  K  P  L  E  Q  V  A  W  L  K  P  K  V  E  D  I
421 TTTAATAAATGCTTGGATGGTCTACCTACGAAAGTACCAGAGAATCAATGCAATGACATT
141  F  N  K  C  L  D  G  L  P  T  K  V  P  E  N  Q  C  N  D  I
481 GGCATGACGATTGGACATTGTATTTGGAAAGAAATACAACTGGAATGTCCAAAGGATAGA
161  G  M  T  I  G  H  C  I  W  K  E  I  Q  L  E  C  P  K  D  R
541 CAAGCAAATCTTGAAAAATGTGAGGCGCTTCAAGTATATTTGAAGGAGAACAAGGCTTTC
181  Q  A  N  L  E  K  C  E  A  L  Q  V  Y  L  K  E  N  K  A  F
601 CCACCACCACCACCTATACGTTGTTAG
201  P  P  P  P  P  I  R  C
```

Figure S1. Nucleotide sequence and deduced amino acid sequence of HparOBP14 from *H. parallela*. The signal peptide is represented by a single underscore, and the start codon and stop codon are represented by a rectangle.

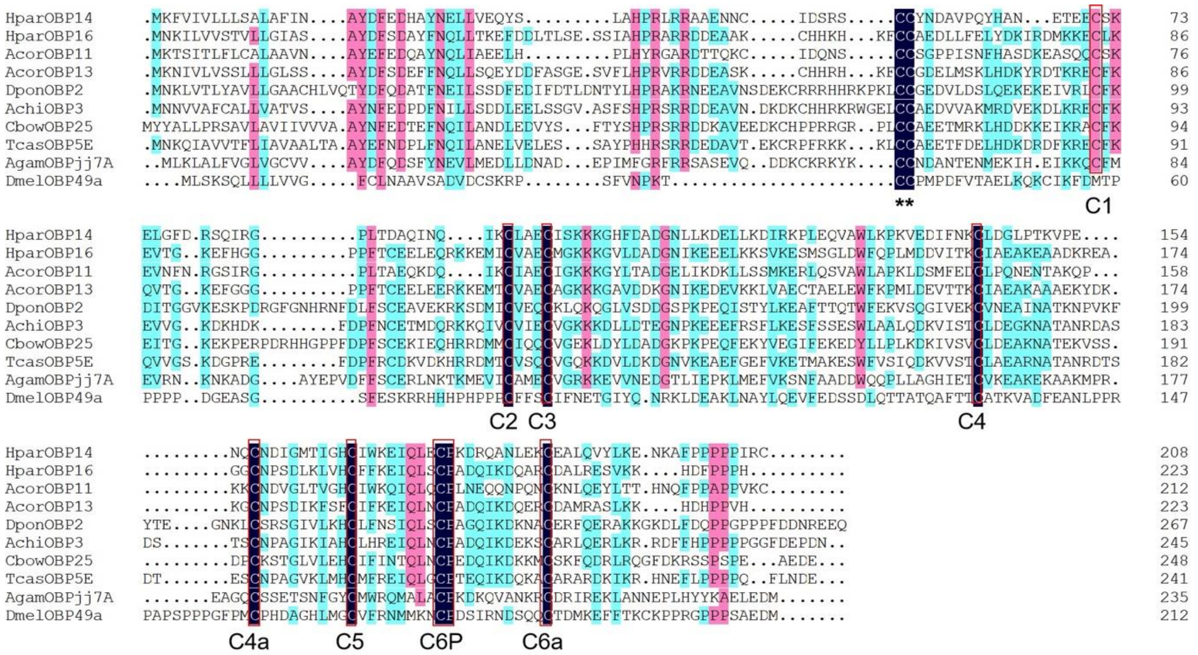

Figure S2. Alignment of the deduced amino acid sequence of HparOBP14 with homologous proteins from other OBPs. The eight conserved Cys residues are labelled by 'C'. ‘*’ indicates the conserved cysteine residue before C1. The species are *Anomala corpulenta* (Acor), *Anoplophora chinensis* (Achi), *Dendroctonus ponderosae* (Dpon), *Tribolium castaneum* (Tcas), *Colaphellus bowringi* (Cbow), *Anopheles gambiae* (Agam) and *Drosophila melanogaster* (Dmel). Their GenBank accession numbers are AcorOBP11 (AKC58532.1), AcorOBP13 (AKC58534.1), HparOBP16 (AKI84374.1), AchiOBP3 (AUF72950.1), CbowOBP25 (ALR72513.1), DponOBP2 (AKK25130.1), TcasOBP5E (EFA03231.1), AgamOBPjj7A (EAA43809), DmelOBP49a (NP_ 610812.1).

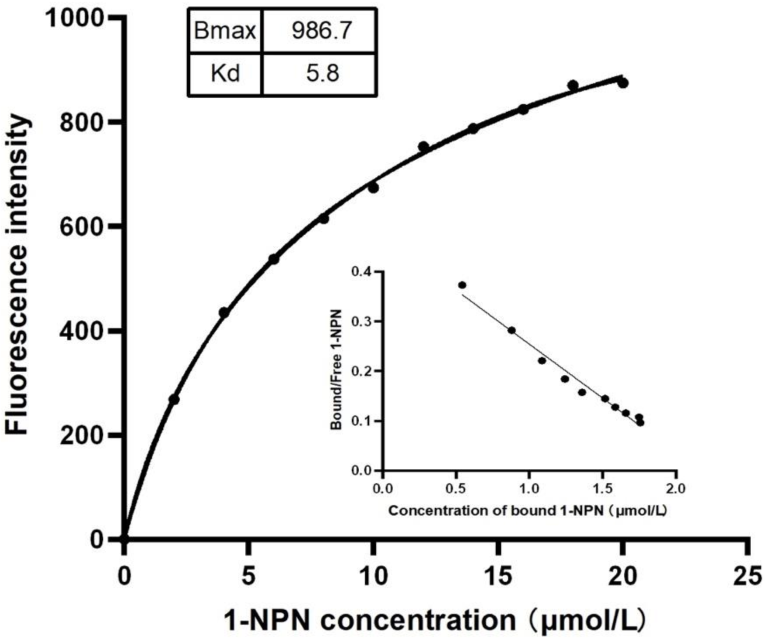

Figure S3. Binding curve and Scatchard plots of HparOBP14 protein with fluorescent probe 1-NPN.
